# Challenge of tracheal extubation and tube exchange of a difficult airway: a narrative review

**DOI:** 10.1007/s00540-025-03510-0

**Published:** 2025-05-08

**Authors:** Daniel Heisenberg, Andrea Stieger, Frank Oberle, Matteo Parotto, Thomas Heidegger

**Affiliations:** 1 HOCH Health Ostschweiz, Kantonsspital St. Gallen, Klinik Für Anästhesiologie, Schmerz- Und Rettungsmedizin, 9007 St. Gallen, Switzerland; 2 HOCH Health Ostschweiz, Spital Grabs, Klinik Für Anästhesiologie, Intensiv- Und Rettungsmedizin, Spitalstrasse 44, 9472 Grabs, Switzerland; 3 Department of Anesthesia and Pain Management, Toronto General Hospital, Toronto, Canada; 4 Department of Anaesthesiology and Pain Medicine, Inselspital, Bern University Hospital, University of Bern, 3012 Bern, Switzerland

**Keywords:** Airway management, Difficult airway, Extubation, Tube exchange

## Abstract

Tracheal extubation is an integral part of airway management. Even though available data indicated that the incidence of complications immediately after tracheal extubation may be higher than during tracheal intubation, it is significantly underexplored in the scientific literature in comparison with tracheal intubation. Failure to re-secure the airway during or immediately after tracheal extubation may have fatal consequences. Closed claims analyses have highlighted the seriousness of adverse events occurring postextubation. Consequently, a well-planned strategy for tracheal extubation is as important as for the intubation and is correctly regarded as a logical extension of an intubation strategy. This narrative review focusses on the challenges of tracheal extubation and complications of routine and ‘at-risk’ extubation in adults. It provides the reader with a risk stratification before extubation. Guidelines for tracheal extubation including advanced techniques for tracheal extubation of patients ‘at-risk’ are followed by a detailed step-by-step approach for video-assisted tracheal tube exchange in patients with a difficult airway.

## Introduction

Airway management is a fundamental component of patient safety in the perioperative environment as well as in the care of the critically ill, encompassing the whole process from securing the airway until extubation [1]. Interestingly, however, tracheal extubation generates less interest than laryngoscopy and/or tracheal intubation and is significantly underexplored in the scientific literature in comparison with tracheal intubation [2–4]. Minor issues such as coughing and breath-holding which normally do not have a relevant impact on patients’ outcome are common, whereas more serious complications such as airway obstruction or pulmonary aspiration are rare and often preventable with proper planning [1]. Consequently, a well-planned strategy for tracheal extubation is as important as for the tracheal intubation and is correctly regarded as a logical extension of an intubation strategy [1, 5]. Reintubation of a failed tracheal extubation or tracheal tube exchange, especially in an emergency are often more challenging and may fail due to physiological instability (e.g., increased oxygen demand, decreased oxygen reserve, hemodynamic instability), anatomical changes, restricted airway access, or due to lack of a clear strategy in combination with a lack of experienced personnel [1, 6]. This narrative review focusses on the challenges of extubation and complications of routine and ‘at-risk’ extubation in adults. It provides the reader with a risk stratification before extubation. Guidelines for tracheal extubation including advanced techniques for extubation of patients ‘at-risk’ are followed by a detailed step-by-step approach for video-assisted tracheal tube exchange in patients with difficult airways.

## Prevalence of problems at extubation

In general, the incidence of respiratory complications immediately after tracheal extubation is higher than during tracheal intubation [7]. Complications immediately after tracheal extubation occurs in almost 13% and in the recovery room in almost 10% of cases [7]. In a study including over 24,000 patients, hypoxemia (Sp02 < 90%) was identified as the most common cause of critical postoperative respiratory events [8]. Failed tracheal extubation (i.e., the necessity to re-intubate shortly after) occurs in approximately 0.06%–0.1% in adults who underwent tracheal intubation for general anesthesia [9, 10]. The prevalence increases roughly tenfold for patients having procedures involving their airway, for patients extubated in critical care areas and in patients with obstructive sleep apnea [1]. Failure to re-secure the airway may have fatal consequences. Clinical surveys and closed claims analyses have highlighted the seriousness of adverse events occurring post-extubation [7, 11, 12]. The Fourth National Audit Project (NAP4) in the United Kingdom reported that 28% of serious complications, including brain injury and death occurred at emergence, or following extubation [13]. The American Society of Anesthesiologists (ASA) Closed Claims database found that 18% of death and brain damage arising from management of the difficult airway occurred during or after tracheal extubation [11]. Most of the claims were associated with an anatomically difficult airway, obesity or obstructive sleep apnea.

## Challenge of tracheal extubation

### Complications of tracheal extubation

The overall risk of any extubation relates to the interaction between the risks of tracheal extubation being tolerated, and if reintubation is required, the probability that it can be accomplished safely [1]. Both of them having an element of uncertainty. Tracheal extubation is normally a planned procedure and removal of a tracheal tube is usually uneventful. However, even routine tracheal extubations may be associated with complications, such as hypertension, tachycardia, or coughing (Table 1) [14, 15].

**Table 1:** Complications of tracheal extubation

Complication	Surgical and medical setting
Miscellaneous	Unintended extubation Hypertension, tachycardia Increased intracranial pressure Increased intraocular pressure Coughing, breath-holding Laryngeal injury Laryngospasm or vocal cord paralysis Stridor, airway obstruction Postobstructive pulmonary edema Laryngeal incompetence Aspiration
Airway obstruction	Laryngeal edema Postextubation stridor Laryngospasm Macroglossia Laryngeal or tracheal injury Paradoxical vocal cord motion Postobstructive pulmonary edema (negative pressure pulmonary edema) After thyroidectomy, anterior cervical surgery, or carotid artery surgery: Wound swelling, hematoma Vocal cord dysfunction (eg, recurrent laryngeal nerve injury) Hypoglossal nerve injury Maxillofacial or nasopharyngeal trauma Obesity, morbid obesity, and obstructive sleep apnea Rheumatoid arthritis Parkinson disease Prolonged intubation
Inadequate ventilation	Increased work of breathing (decreased compliance/increased resistance) Diaphragmatic splinting Central hypoventilation syndrome or obstructive sleep apnea Severe chronic obstructive pulmonary disease Residual sedation or neuromuscular blockade Preexisting neuromuscular disorder Relative hypoventilation (e.g., increased CO2 production)
Inadequate oxygenation	Inadequate inspired oxygen concentration Ventilation-perfusion mismatch Right-to-left shunt Increased oxygen consumption Decreased oxygen delivery (mixed venous desaturation) Impaired pulmonary diffusion
Failure of pulmonary toilet	Obtundation Pulmonary secretions Increased volume of secretions Inspissated secretions Impaired mucociliary clearance Neuromuscular impairment
Inability to protect airway	Obtundation Neuromuscular disorder

Adapted from Cooper RM, Parotto M. Extubation and reintubation of the difficult airway. In: Hagberg and Benumof's Airway Management. 5th ed. Elsevier 2023. p.853–75. With permission

In patients with a known difficult airway or in situations, where the airway (anatomically) or the patient’s situation (physiologically) during surgery or prolonged intubations has “changed”, e.g., due to laryngeal edema, multiple intubations attempts, wound swelling, preexisting hypoxia or increased oxygen consumption, the risk for arising complication is generally increased (see below) [16–19].

Finally, it may result in the inability to tolerate tracheal extubation and the requirement for reintubation (Table 1).

#### Unplanned tracheal extubation

The incidence of unplanned tracheal extubation, both accidental and self-extubation as reported in the literature varies widely from a median of 7.3% (0.5–35.8%) in adults to as high as 18.2% (1–80.8%) in the neonatal population [20].

The majority of studies are from the intensive care units (ICU). A recent prospective multi-center study in adult patients from France evaluated the incidence of unplanned tracheal extubation in intensive care units [21]. During the 12-month inclusion period, they found a pooled incidence of 1.0 per 100 mechanical ventilation days (88% were self-extubations and 12% accidental intubations). The incidence of unplanned tracheal extubation in the operating room is unknown but is very likely less common than in the ICU. Unplanned tracheal extubation by patients themselves or during positioning maneuvers can result in significant harm or death [22]. The placement of the patient in prone position (for example in the operating room for surgical procedure needs, or in ICU environments as part of the management of acute respiratory distress syndrome) increases the risk of unintentional tracheal extubation [23]. In addition, most of these patients are in a critical status of oxygenation and usually require immediate reintubation.

#### Airway obstruction

The NAP4 report found that airway obstruction was the primary cause of all airway complications at the end of anesthesia and in the post anesthesia care unit [13]. A patent airway is a prerequisite for successful tracheal extubation but there is a high risk of airway obstruction during emergence [3]. The inability of a patient to protect the airway is often the result of a residual neuromuscular blockade following incomplete antagonism of neuromuscular blocking agents and most commonly presents as airway obstruction. A meta-analysis demonstrated that patients being transferred to post anesthesia care unit are frequently observed with residual neuromuscular blockade, 12% have a train-of-four ratio of < 0.7 and 41% < 0.9 [24]. In principle, the use of neuromuscular blocking agents during general anesthesia is associated with an increased risk of postoperative pulmonary complications [25]. The causes of airway obstruction during emergence and tracheal extubation are shown in Table 2.

**Table 2:** Patient-related, surgical and anesthetic factors that contribute to airway obstruction during emergence and extubation

**Patient-related factors**	
	Obesity
	Obstructive sleep apnoea
	Smoker
	C-spine immobilit
	History of head and neck radiotherapy
	Pharyngeal obstruction (tonsillar/adenoidal hypertrophy)
	Craniofacial abnormalities (micrognathia, maxillary hypoplasia)
	Neuromuscular disorders (bulbar weakness)
	Connective tissue disorders
	Storage disease
	Chronic renal failure
	Laryngomalacia
**Surgical factors**	
	Airway soiling (blood, secretions)
	Swelling
	Vocal cord damage
	Neck haematoma
	Trendelenburg position (facial and airway oedema)
	Fixation of cervical spine or facial bones (causes reduced head and neck mobility)
**Anesthetic factors**	
	Anesthetic agents (reduce consciousness, impair reflexes, reduce muscle tone)
	Laryngospasm
	Residual neuromuscular blockade
	Glottic oedema
	Airway device occlusion (from biting, secretions or blood)

From Benham-Hermetz J, Mitchell V. Safe tracheal extubation after general anaesthesia. BJA Education 2021; 21: 446–54. With permisson

#### Post-obstructive pulmonary edema

Post-obstructive pulmonary edema, also referred to as negative pressure pulmonary edema, can develop after an episode of airway obstruction [1, 3, 14, 26]. It was recognized in 10% of all anesthesia-related airway complications in the NAP4 study [13]. It is caused by a forced inspiratory effort against a closed glottis or an occluded airway. This generates a negative intrathoracic pressure that alters the Starling forces across the pulmonary capillaries and alters cardiac filling pressures and afterload. The result is movement of fluid into the alveoli and pulmonary interstitium with pulmonary oedema despite normal cardiac function [3]. The diagnosis should be suspected when tachypnea, cough, pink sputum, hypoxia, and diffuse pulmonary infiltrates in the chest radiographs are observed following the relief of upper airway obstruction. Management is supportive with oxygen, application of continuous positive airway pressure and diuretics when appropriate [3, 15].

#### Laryngospasm

Laryngospasm is a common complication of general anesthesia and results from direct irritation of the vocal cords by blood, saliva or instrumentation, or indirectly from surgical stimulation [3, 15, 26]. Laryngospasm can best be prevented by tracheal extubation at a sufficiently deep plane of anesthesia or awaiting full recovery of consciousness [1]. In this regard, ensuring sufficient depth of anesthesia before manipulation of the airway, removal of airway blood and secretions are important [3].

## Risk stratification before planned tracheal extubation

**Fig. 1:**
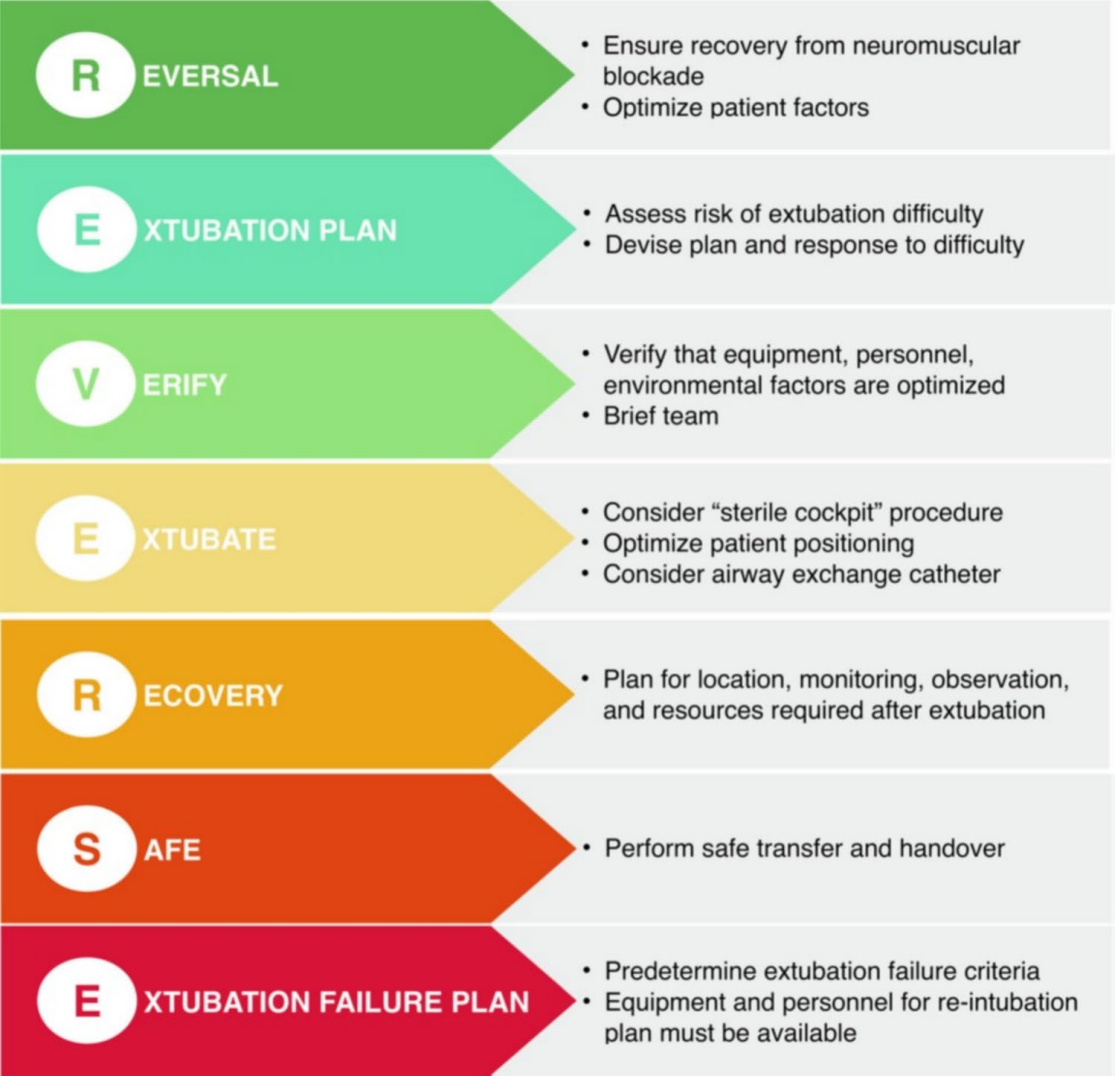
Canadian Airway Focus Group updated consensus-based recommendations for management of the difficult airway: considerations for planning for safe tracheal extubation (“REVERSE”). From Law JA, Duggan LV, Asselin M, Baker P, Crosby E, Downey A, et al. Canadian Airway Focus Group updated consensus-based recommendations for management of the difficult airway: part 2. Planning and implementing safe management of the patient with an anticipated difficult airway. Can J Anesth. 2021; 68:1373–1404. With permission

In general, patients should be hemodynamically stable and demonstrate adequate oxygenation and ventilation prior to tracheal extubation. In 2021, the Canadian Airway Focus Group in their updated consensus-based recommendations for management of the difficult airway [27] described principal considerations for planning for safe tracheal extubation (“REVERSE”) (Fig. 1).

Obesity, defined as a BMI greater than or equal to 30 kg/m2 is the most important preexisting medical conditions that might compromise safe tracheal extubation. In 2022, 43% of adults aged 18 years and over were overweight and 16% were living with obesity (https://www.who.int/news-room/fact-sheets/detail/obesity-and-overweight). Obese patients are at risk of having undiagnosed obstructive sleep apnea which can worsen perioperative complications due to inadequate pre- or postoperative management [28]. Patients with obesity are more prone to complications, such as quick oxygen desaturation, challenges with airway management, increased gastric reflux, and potential airway blockages [6, 29]. A meta-analysis, including 13 studies, has shown that patients with obstructive sleep apnea syndrome suffer more likely from postoperative respiratory failure (odds ratio 2.4), and more likely to require a reintubation in their perioperative course (odds ratio 2.1) compared to non-obese individuals [30].

### Deep versus awake tracheal extubation

In principle, tracheal extubation may be performed before (“asleep” tracheal extubation) or after recovery of consciousness [31]. In a fully awake patient, sufficiently spontaneously breathing, the patient is able to protect and maintain a patent airway [3]. The purported advantage of deep tracheal extubation is avoidance of the adverse reflexes associated with (awake) tracheal extubation, such as coughing, hypertension, and laryngospasm or an increase of intraocular and intracranial pressure [1]. However, deep or asleep tracheal extubation is not appropriate for patients at risk of aspiration and in whom bag-mask ventilation or reintubation would be challenging [3, 31]. In a prospective study on 1005 patients, complications were more likely if the trachea was extubated, while the patient was still deeply anesthetized rather than after the patient had regained consciousness, regardless of the type of surgery [7].

## Extubation guidelines

**Fig. 2:**
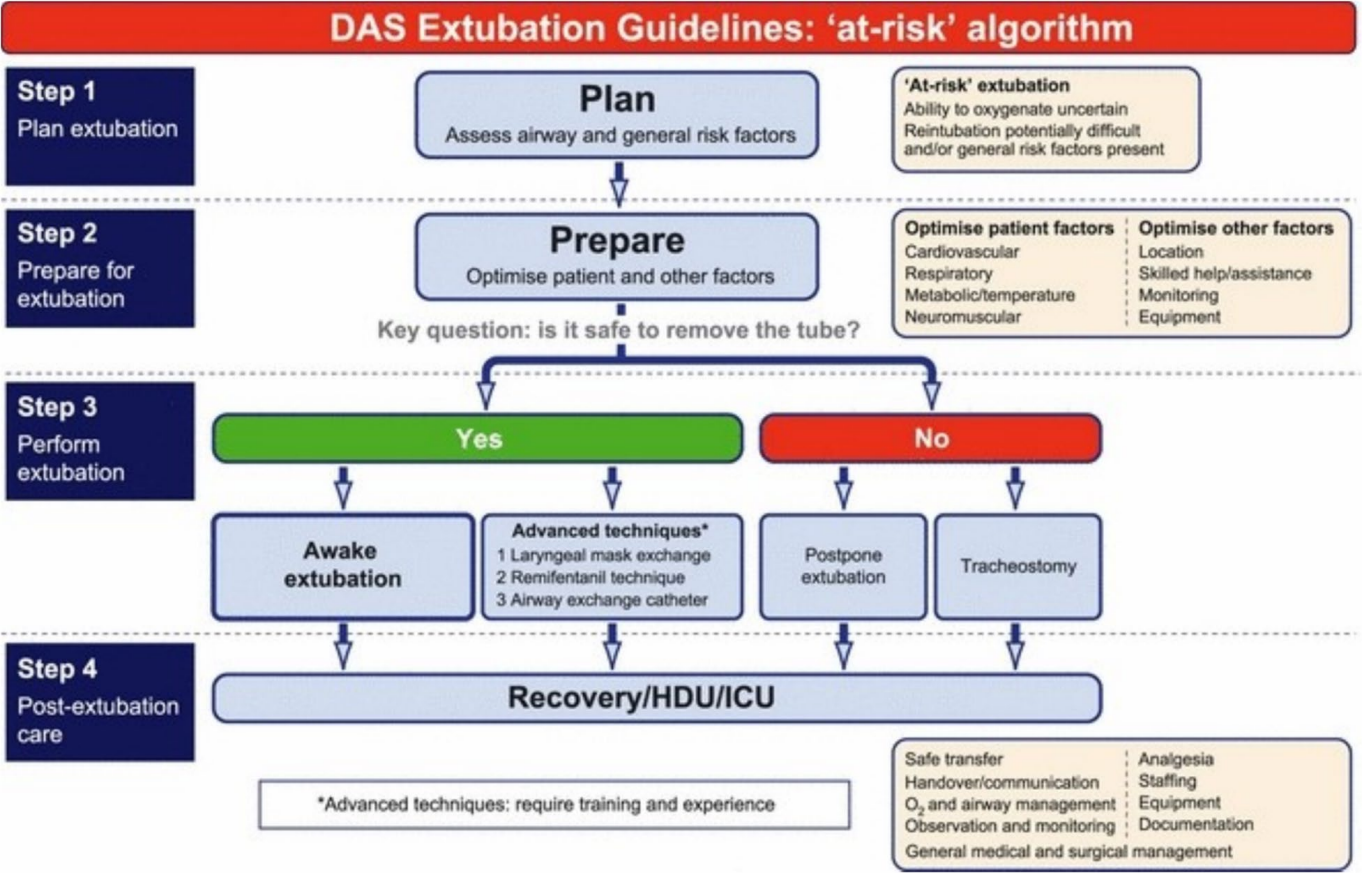
Difficult Airway Society extubation guidelines: ‘at-risk’ algorithm. From Popat M, Mitchell V, Dravid R, Patel A, Swampillai C, Higgs A. Difficult Airway Society Guidelines for the management of tracheal extubation. Anaesthesia 2012; 67:318–40. With permission

As initially described, tracheal extubation is an integral part of (difficult) airway management. As a consequence, in recent years, various international scientific airway management societies have also included or have developed guidelines for this scenario [5, 27, 32–36]. A step-by-step process (preformulated strategy) is elucidated in the DAS guidelines [33]. *Step 1* Develop a plan for tracheal extubation in which an airway assessment is performed and general risk factors are acknowledged. *Step 2* Prepare for tracheal extubation by optimizing the patient and any risk factors and categorizing the patient as either low or high risk. At-risk patients include those in which the ability to oxygenate is uncertain, reintubation is potentially difficult, and/or general risk factors, such as specific surgical requirements or medical conditions, are present. *Step 3* Perform the tracheal extubation using the “low risk” or “at risk” algorithm. *Step 4* Determination of post-extubation care (recovery room, stepdown unit or intensive care setting). The “at-risk” algorithm of the DAS extubation guidelines is shown in Fig. 2. Once the decision has been made that it is safe to remove the tube, awake tracheal extubation or tracheal extubation using an advanced technique can be performed (see below).

### Advanced techniques in patients at risk

In high-risk cases, where tracheal extubation might not be tolerated or reintubation difficulty is predicted, some additional techniques can help to ensure tracheal extubation success. In patients at risk of airway edema due to significant positive fluid balance, prolonged prone or Trendelenburg position, or following neck, cervical and face surgery, a cuff leak test or ultrasonography can be performed to assess readiness. A successful cuff leak test means that there is an air leak around the tube after deflating the balloon cuff of the tracheal tube [37]. A cuff leak test prior to tracheal extubation, however, has limited diagnostic power with moderate sensitivity and good specificity which means that the test works better to rule out potential post-extubation airway obstruction than in identifying patient at risk for post-extubation problems [38–40].

Ultrasonography can be used to measure the air column width which is defined as the width of the acoustic shadow present at the level of the vocal cords [40]. If the air column width is measured before and after tracheal cuff deflation, the air column width difference can be calculated. Ultrasonography has a higher sensitivity and specificity compared to the cuff leak test to predict post-extubation stridor [9, 39–41]. However, these findings should also be interpreted with caution, since available evidence is limited to small-scale studies [40, 42].

If a cuff leak exists and there are no other disturbing airway issues, such as inadequate oxygenation or spontaneous breathing, predictors of a difficult airway or impaired airway reflexes, tracheal extubation can proceed.

In the absence of a cuff leak, for example due to post-extubation laryngeal edema [40], tracheal extubation should be delayed, and consideration should be given to administering steroids to reduce the edema and increase the likelihood of successful tracheal extubation [1].

In high-risk cases, airway exchange catheters may be utilized as a ‘place-holder’ to facilitate reintubation, should this be required. In such fashion, an airway exchange catheter would be inserted via the tracheal tube at depth aligned with the distal end of the tracheal tube, prior to tracheal tube removal. The airway exchange catheter is then left in place in the trachea and the patient is closely monitored. The device would be left in situ until there is no longer concern about requiring early reintubation. Properly inserted (tip above the carina), they are normally well-tolerated in awake patients. The most used airway exchange catheter is from Cook [1, 43], which is available in different sizes and lengths.

It is also possible to use a tracheal extubation set specifically designed for high risk extubations, where a flexible guidewire can be left in place after tracheal extubation. If reintubation is necessary, a soft tipped airway exchange catheter can be passed over the guidewire and a new tracheal tube can be advanced over the airway exchange catheter. Airway exchange catheters have a high rate of success when used as a guide to reintubate [44]. Success rate can be further improved using video laryngoscopy to facilitate accurate positioning and to avoid tube impingement [45]. If reintubation fails, a forward strategy injecting a neuromuscular blocking agent and preparing for an emergency cricothyrotomy must be considered [1]. In complex cases, such as those involving ear, nose and throat surgery with flap creation, potential airway swelling, or compromised airway integrity, tracheal extubation may be unsafe even if delayed, and elective tracheotomy should be considered to ensure patient safety [27].

## Video-assisted tube exchange of a patient with a difficult airway

**Fig. 3:**
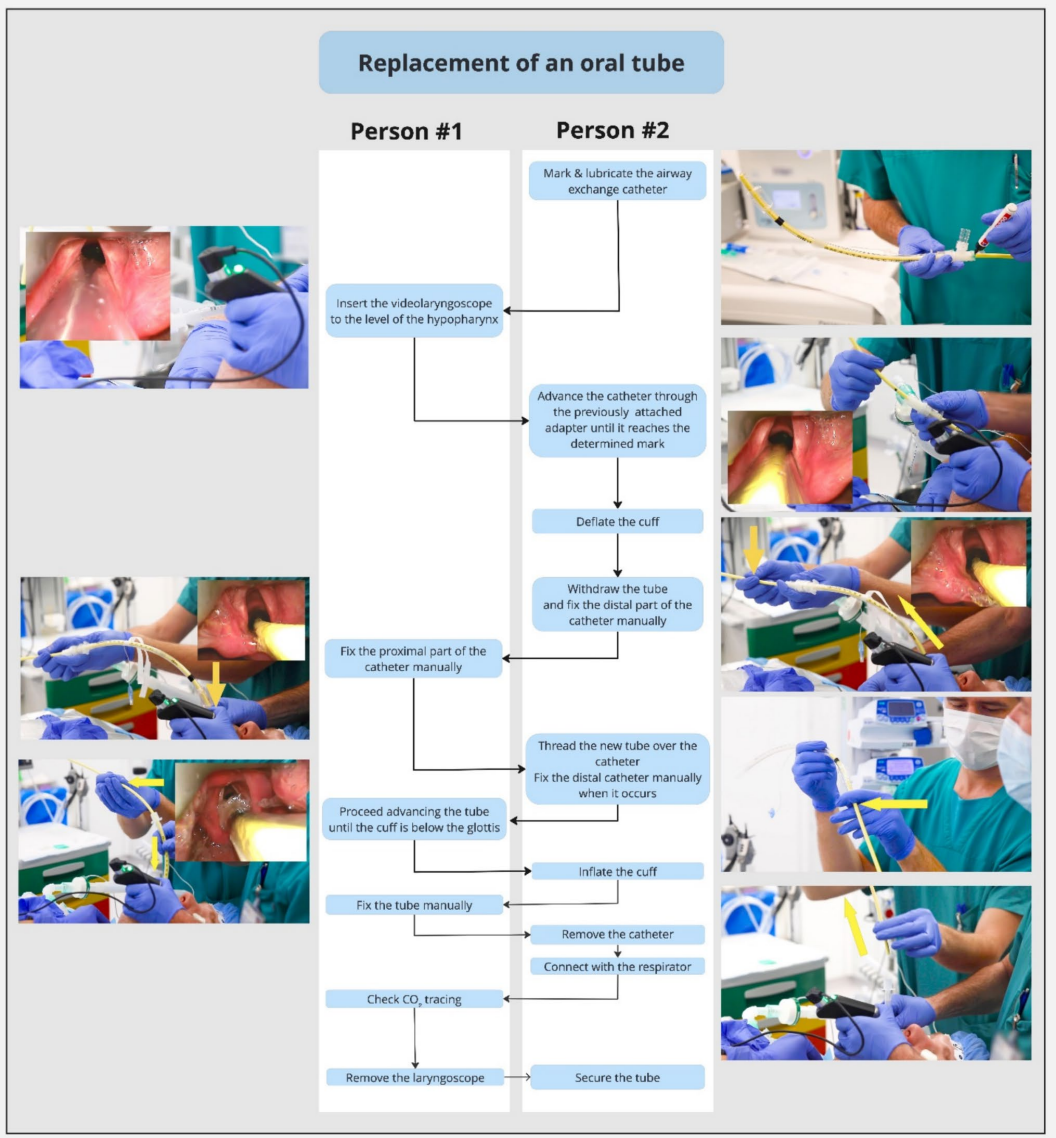
Flow-chart video-assisted replacement an oral tube with a new oral tube. Adapted from Heidegger T, Oberle F. Endotracheal tube exchange. N Engl J Med. 2023; 25;388(21):e71. https://doi.org/10.1056/NEJMc2304779. With permission

**Fig. 4:**
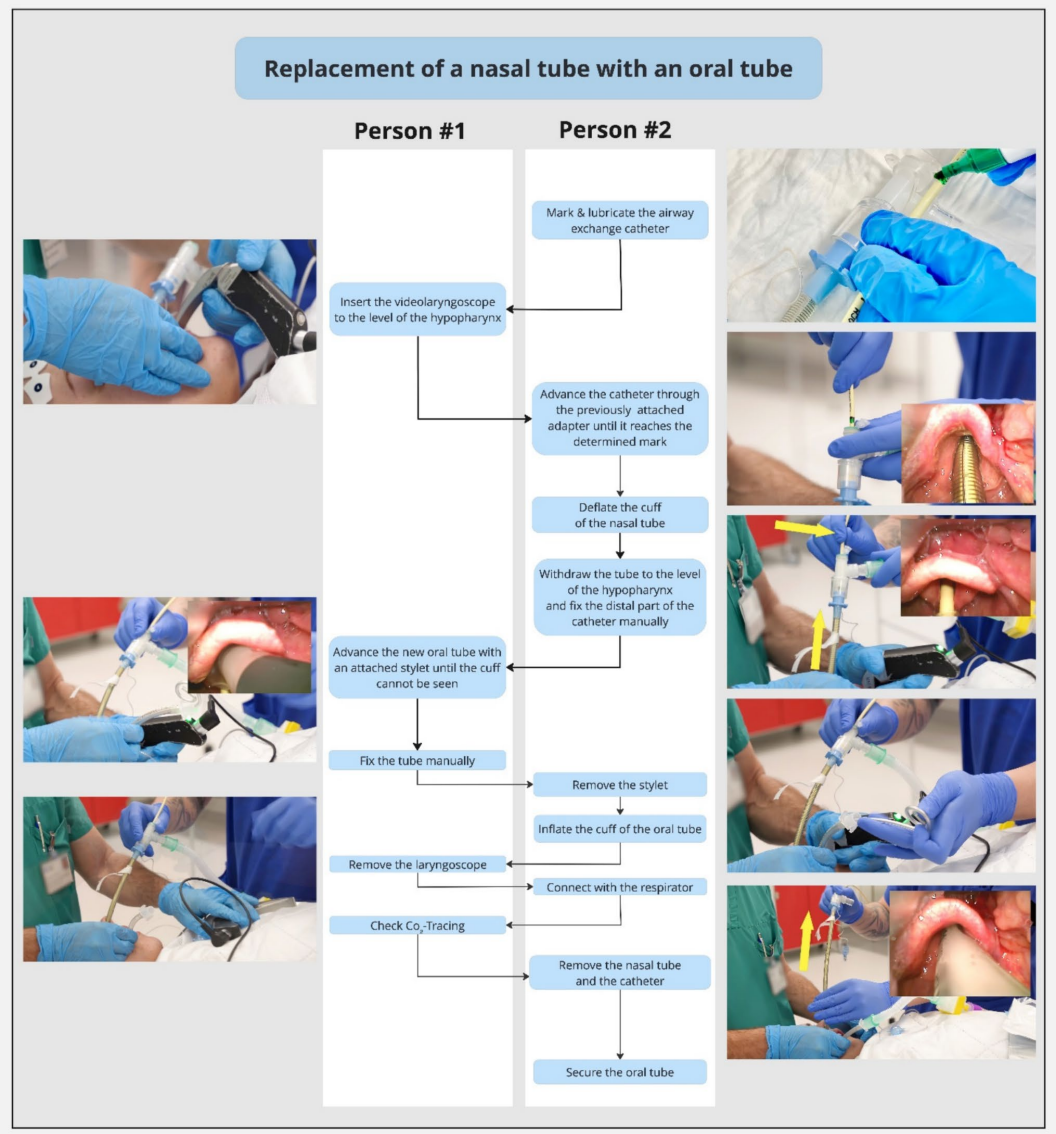
Flow-chart video-assisted replacement a nasal tube with an oral tube. Adapted from Heidegger T, Oberle F. Endotracheal tube exchange. N Engl J Med. 2023; 25;388(21):e71. https://doi.org/10.1056/NEJMc2304779. With permission

Video-assisted tracheal tube exchange is indicated when the tube that is currently in place has a broken cuff, is too small and a larger tube is required, needs to be switched from a single-lumen to a double-lumen tube or vice versa, or when a nasal tube needs to be replaced with an oral tube [46]. Figures 3 and 4 show a detailed step-by-step guidance how to replace an oral tube with a new oral tube and the replacement of a nasal tube with an oral tube, thereby exactly allocating the tasks of the involved persons. The most common problem that occurs during tube exchange is inadvertent removal of the airway exchange catheter and consequent “loss” of the airway [46]. However, when the entire procedure is performed under direct visualization, this complication can usually be prevented. If oxygen saturation is decreasing during the procedure, stop the procedure. In the case of a nasal tube exchange, replace the original nasal tube over the airway exchange catheter; in the case of an oral tube exchange, replace it with a smaller tube [46].

Oxygen administration through an airway exchange catheter (especially via jet ventilation but also through insufflation) should be avoided as this may be associated with a risk of barotrauma if the tip of the catheter becomes accidentally placed in a bronchus and causes occlusion [27, 43]. Should a patient decompensate with an airway exchange catheter in situ, tracheal reintubation is the key management strategy. Supplemental oxygen can be provided using standard techniques prior to tracheal intubation or between attempts.

If reintubation has failed, ventilation with a face-mask or a supraglottic airway should be established immediately. In case of a cannot ventilate, cannot oxygenate situation, an emergency front-of-neck-access (cricothyrotomy) must be performed without delay [47].

## Conclusion

Managing tracheal extubation in a patient with a difficult airway is an integral part of safe airway management and requires careful planning, timely intervention, and the anticipation of complications based on patient-specific and procedural factors. Risk stratifications and the use of advanced techniques for reintubation such as the use of an airway exchange catheter must be considered. Video-assisted tracheal tube exchange with the use of an airway exchange catheter is an important part of difficult airway management that should be performed only by physicians experienced with this technique. Future research should focus on refining these strategies to further enhance safety and outcomes in airway management.
